# Enhanced Channel Estimation for RIS-Assisted OTFS Systems by Introducing ELM Network

**DOI:** 10.3390/s25113292

**Published:** 2025-05-23

**Authors:** Mintao Zhang, Zhiying Liu, Li Wang, Wenquan Hu, Chaojin Qing

**Affiliations:** School of Electrical Engineering and Electronic Information, Xihua University, Chengdu 610039, China; zhangmt@mail.xhu.edu.cn (M.Z.); liuzhiying@stu.xhu.edu.cn (Z.L.); wang_li@stu.xhu.edu.cn (L.W.); huwenquan@stu.xhu.edu.cn (W.H.)

**Keywords:** channel estimation, symbol detection, extreme learning machine, orthogonal time frequency space, reconfigurable intelligent surfaces

## Abstract

In high-mobility communication scenarios, leveraging reconfigurable intelligent surfaces (RISs) to assist orthogonal time frequency space (OTFS) systems proves advantageous. Nevertheless, the integration of RIS into OTFS systems increases the complexity of channel estimation (CE). Utilizing the benefits of machine learning (ML) to address such intricate issues holds the potential to reduce CE complexity. Despite this potential, there is a lack of investigations of ML-based CE in RIS-assisted OTFS systems, leaving significant gaps and posing challenges for intelligent applications. Moreover, ML-based CE methods encounter numerous difficulties, including intricate parameter tuning and long training time. Motivated by the inherent advantages of the single-hidden layer feed-forward network structure, we introduce extreme learning machine (ELM) into RIS-assisted OTFS systems to improve CE accuracy. In this method, we incorporate a threshold-based approach to extract initial features, aiming to remedy the inherent limitations of the ELM network, such as inadequate network parameters compared to the deep learning network. This initial feature extraction contributes to an enhanced ELM learning ability, leading to improved CE accuracy. Applying the classic message passing algorithm for data symbol detection, simulation results demonstrate the effectiveness of the proposed method in improving the symbol detection (SD) performance of RIS-assisted OTFS systems. Furthermore, the SD performance exhibits its robustness against variations in modulation order, maximum velocity, and the number of sub-surfaces.

## 1. Introduction

### 1.1. OTFS in High-Mobility Scenarios

The key performance requirements in next-generation wireless communication include high mobility, increased energy and spectral efficiency, high reliability and low latency [1,2,3]. In high-mobility communication scenarios, orthogonal time frequency space (OTFS) modulation has been widely considered to ensure reliability [4,5,6,7]. However, frequent communication disruptions caused by physical obstructions in wireless scenarios remain a critical issue [8,9,10].

### 1.2. The Integration of RIS with OTFS Modulation

Reconfigurable intelligent surfaces (RISs) have emerged as a highly promising solution to mitigate physical blockages by intelligently controlling the wireless propagation environment [11,12,13]. Therefore, a combination of RIS and OTFS offers comprehensive advantages, including flexible channel configuration and reliable communication in high-mobility scenarios. Recent works highlight the effectiveness of deploying RIS in OTFS systems to enhance overall system performance. In [14], it is demonstrated that RIS-assisted OTFS systems outperform RIS-assisted OFDM systems, exhibiting excellent performance over a wide range of channel parameters. An efficient and reliable transmission scheme is proposed in [15], which leverages the information in the Doppler-delay (DD) domain to facilitate the configuration of RIS. In [16], efficient CE and symbol detection (SD) performance is achieved for hybrid RIS-assisted millimeter wave OTFS systems by combining the expectation maximization with the message passing (MP) algorithms.

### 1.3. CE Challenges and ML Methods

Channel estimation (CE) is a critical aspect of receiver design in RIS-assisted OTFS systems. However, the introduction of RIS in OTFS systems presents a great challenge of CE in terms of processing complexity. On the one hand, the large number of passive reflective elements in RIS results in a tremendously large channel matrix dimension [17,18,19]. On the other hand, the absence of signal processing capability in the passive components of RIS exacerbates the processing complexity [20]. Existing works have been researched on CE in RIS-assisted OTFS systems [15,16]. However, the challenge of CE complexity in RIS-assisted OTFS systems still requires further exploration. Leveraging the significant advantages of machine learning (ML) in addressing complex problems, the introduction of ML into RIS-assisted OTFS systems holds the promise of overcoming this CE challenge. Deep learning (DL) has demonstrated significant potential in improving the performance of communication systems through data-driven CE and adaptive network optimization [21,22,23,24]. In particular, preliminary efforts have explored DL-based CE methods for RIS-assisted systems. In [25], a super-resolution convolutional neural network (CNN) and a denoising CNN are proposed for CE in an RIS-aided multi-user multiple-input multiple-output (MIMO) orthogonal frequency division multiplexing (OFDM) system. In [26], a twin CNN is proposed for CE in RIS-assisted massive MIMO systems. In [27], CE is modeled as a denoising problem, and a deep residual learning-based neural network is developed for RIS-assisted multi-user communication systems. In [28], a convolutional blind denoising network, a convolutional denoising generative adversarial network, and a multiple residual dense network are proposed to obtain channel state information for mmWave RIS-aided MIMO systems. For RIS-aided OTFS systems, a dilated attention generative adversarial network is proposed to perform CE in [29]. However, the processing complexity of CE in RIS-assisted OTFS systems has not been effectively addressed. Among numerous ML methods, DL-based approaches face challenges such as intricate parameter tuning, extensive dataset collection, prolonged training times, etc. [30]. In contrast, extreme learning machine (ELM)-based methods exhibit significant advantages, positioning them as an attractive potential solution [31]. Unlike DL-based approaches, the ELM benefits from its single-hidden layer feed-forward neural network structure [31]. This key feature obviates the need for gradient back-propagation (BP) and confers several notable advantages, such as exceptional learning speed (hundreds of times faster than the BP algorithm) and impressive generalization performance [32,33,34].

### 1.4. Motivation and Contributions

Inspired by the advantages of the ELM network, this paper introduces ELM into the CE of RIS-assisted OTFS systems. Specifically, recognizing the inherent limitations of ELM, e.g., the inadequate network parameters relative to DL networks, we integrate a threshold-based method to extract initial features. This processing simplifies the channel representation in the DD domain and facilitates the learning of comprehensive channel features. Subsequently, we develop an ELM network to improve CE accuracy according to the extracted initial features. By leveraging the enhanced channel state information, we recover data symbols using the classic MP algorithm [6]. The proposed method efficiently facilitates the CE in high-mobility communication scenarios, thereby improving subsequent SD performance. The main contributions of this paper are as follows:

1. We propose an enhanced CE method for RIS-assisted OTFS systems by introducing an ELM network. To the best of our knowledge, few works have exploited ELM-based CE for RIS-assisted OTFS systems. Although existing DL-based methods achieve effective CE in RIS-OTFS systems (e.g., [29]), the high processing complexity motivates the exploration of alternative methods to alleviate challenges such as intricate parameter tuning and long training time.
2. To mitigate the limited learning capability of ELMs relative to deep networks, we incorporate a threshold-based feature extraction method that enhances input representation despite the reduced model complexity. This initial feature extraction takes into account the specificity of CE in OTFS systems, setting it apart from current ELM-based CE methods (e.g., [32]).
3. We incorporate the cascaded channels of RIS into the representation of the DD domain equivalent channels of the OTFS systems. This enables the simplification of CE in high-mobility communication scenarios by leveraging the approximately time-invariant channel characteristics in the DD domain [35], and thus improves the CE accuracy of RIS-assisted OTFS systems [16].

The rest of this paper is organized as follows. Section 2 illustrates the system model of RIS-assisted OTFS. In Section 3, an enhanced CE method introducing an ELM network is proposed. Section 4 provides numerical simulation results and performance analysis. Finally, we conclude the paper in Section 5.

**Notation 1.**  *Bold face lowercase and uppercase letters represent vector and matrix, respectively. ${\left(\;\cdot \;\right)}^{T}$ and ${\left(\;\cdot \;\right)}^{H}$ denote the transpose and conjugate transpose, respectively. ⊙ stands for the Hadamard product. $F_{M}$ and $F_{N}$ are the normalized M×M and N×N Fourier transform matrix, respectively. $\mathrm{vec}\left(\;\cdot \;\right)$ and ${\mathrm{vec}}^{-1}\left(\;\cdot \;\right)$ denote the column vectorization and its inverse operation, respectively. $I_{M}$ stands for the M-order unit matrix.*

## 2. System Model

**Figure 1:**
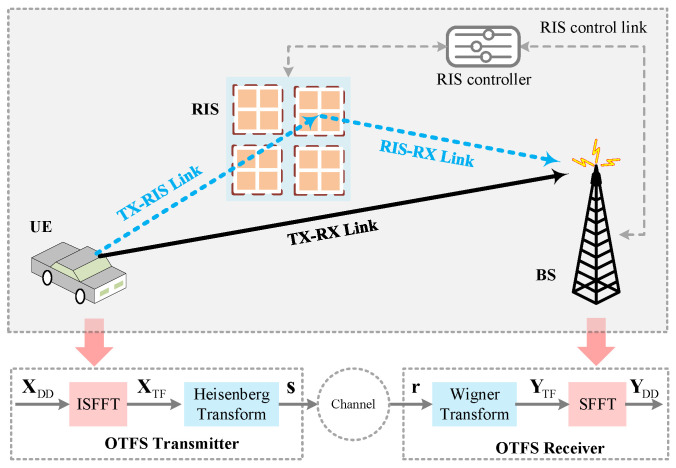
RIS-assisted OTFS systems.

As shown in Figure 1, the single-antenna RIS-assisted OTFS system with *N* delay and *M* Doppler bins is considered in this paper. At the UE transmitter (TX), the information bits are mapped to DD grids to form $X_{\mathrm{DD}}\in C^{M\times N}$. By using the inverse symplectic finite Fourier transform (ISFFT), $X_{\mathrm{DD}}$ is transformed to the time-frequency (TF) domain, which is expressed as [36]

(1) $$X_{\mathrm{TF}}=F_{M}X_{\mathrm{DD}}F_{N}^{H},$$

where $X_{\mathrm{TF}}\in C^{M\times N}$ denotes the TF domain variant of $X_{\mathrm{DD}}$. The Heisenberg transform is employed to transform the TF signal $X_{\mathrm{TF}}$ to the time domain (TD) signal $S\in C^{M\times N}$, i.e.,

(2) $$S=G_{\mathrm{tx}}F_{M}^{H}X_{\mathrm{TF}},$$

where $G_{\mathrm{tx}}\in C^{M\times M}$ denotes the filter operating associated with the pulse-shaping waveform $g_{\mathrm{tx}}\left(t\right)$. $G_{\mathrm{tx}}=\mathrm{diag}\left[g_{\mathrm{tx}}\left(0\right),g_{\mathrm{tx}}\left(\frac{T}{M}\right),\cdots ,g_{\mathrm{tx}}\left(\frac{\left(M-1\right)T}{M}\right)\right]$, and $G_{\mathrm{tx}}=I_{M}$ in the case of rectangular waveforms. Equation (2) can be rewritten as

(3) $$S=F_{M}^{H}X_{\mathrm{TF}}.$$

According to the column-wise vectoring, the TD vector signal $s\in C^{MN\times 1}$ is given by

(4) $$s=\mathrm{vec}\left(S\right).$$

After appending a cyclic prefix (CP), the TD signal is transmitted to the base station (BS) receiver (RX) over the wireless channels.

From [32], we consider the RIS-assisted systems with *R* sub-surfaces to reflect *P* resolvable paths. The composite channel impulse responses (CIRs) between the TX and RX, denoted as $h\in C^{P\times 1}$, are given by [32]

(5) $$h=h_{\mathrm{TR}}+h_{\mathrm{TRR}}\phi ,$$

where $h_{\mathrm{TR}}\in C^{P\times 1}$ denotes the CIRs of the direct TX-RX link, $h_{\mathrm{TRR}}\in C^{P\times R}$ stands for the equivalent cascaded CIRs of the reflecting link (i.e., TX-RIS-RX link), and $\phi \in C^{R\times 1}$ is the phase-shift vector of *R* sub-surfaces. From [32], we express $h_{\mathrm{TRR}}$ and ϕ as

(6) $$h_{\mathrm{TRR}}=\left[h_{\mathrm{TRR},1},h_{\mathrm{TRR},2},\cdots h_{\mathrm{TRR},R}\right],$$

and

(7) $$\phi \overset{\Delta }{=}{\left[\phi_{1},\phi_{2},\cdots ,\phi_{R}\right]}^{T},$$

where $h_{\mathrm{TRR},r}\in C^{P\times 1}$ and $\phi_{r}=\alpha_{r}e^{j\varphi_{r}}$ with r=1,⋯,R denote the equivalent cascaded CIRs and the phase-shift vector of the *r*-th sub-surface, respectively. $\alpha_{r}\in \left[0,1\right]$ and $\varphi_{r}\in \left(0,2\pi \right]$ are the reflection amplitude and the phase shift of the *r*-th sub-surface, respectively. According to [37], we set $\alpha_{r}=1$ and only adjust the phase shift $\varphi_{r}$ to maximize the reflection power and simplify hardware design. For the *r*-th sub-surface, the equivalent cascaded channel $h_{\mathrm{TRR},r}$ is given by

(8) $$h_{\mathrm{TRR},r}=h_{\mathrm{TRS},r}⊙h_{\mathrm{RSR},r},$$

where $h_{\mathrm{TRS},r}\in C^{P\times 1}$ and $h_{\mathrm{RSR},r}\in C^{P\times 1}$ are the equivalent CIRs of the TX-RIS link and RIS-RX link corresponding to the *r*-th sub-surface, respectively.

According to Equations (5) and (8), the composite channel h can be rewritten as

(9) $$h=h_{\mathrm{TR}}+\sum_{r=1}^{R}\left(h_{\mathrm{TRS},r}⊙h_{\mathrm{RSR},r}\phi_{r}\right).$$

For *P* resolvable paths, we have

(10) $$h={\left[h_{1},h_{2},\cdots ,h_{P}\right]}^{T}.$$

Then, the transmission channel $H\in C^{MN\times MN}$ can be represented as [36]

(11) $$H=\sum_{p=1}^{P}h_{p}\Pi^{l_{p}}\Delta^{k_{p}},$$

where $h_{p}$, $l_{p}$, and $k_{p}$ denote the composite channel gain, the normalized delay, and the normalized Doppler shift of the *p*-th (p=1,⋯,P) path. $\Pi \in R^{MN\times MN}$ is the forward cyclic shifted permutation matrix according to delay $l_{p}$ in the DD domain. $\Delta \in C^{MN\times MN}$ models the Doppler shifts in channel, and $\Delta =\mathrm{diag}\left[z^{0},z^{1},\cdots ,z^{MN-1}\right]$ with $z=e^{\frac{j2\pi }{MN}}$. From [5], we have $l_{p}=\left(M\Delta f\right)\tau_{p}$ and $k_{p}=\left(NT\right)\varepsilon_{p}$, where Δf, $\tau_{p}$, *T* and $\varepsilon_{p}$ denote the subcarrier interval, the *p*-th path delay, the sampling interval, and the Doppler shift of the *p*-th path, respectively.

After removing the CP at the receiver, the received TD signal $r\in C^{MN\times 1}$ is given by [36]

(12) r=Hs+n,

where $n\in C^{MN\times 1}$ denotes the complex additive white Gaussian noise vector satisfying $n∼\mathrm{CN}(0,\sigma^{2}I_{MN})$. By using the Wigner transform, the TD signal r is mapped to the TF domain to form $Y_{\mathrm{TF}}\in C^{M\times N}$, which can be expressed as [38]

(13) $$Y_{\mathrm{TF}}=F_{M}G_{\mathrm{rx}}{\mathrm{vec}}^{-1}\left(r\right),$$

where $G_{\mathrm{rx}}\in C^{M\times M}$ stands for the filter matrix in the receiver with the pulse-shaping waveform $g_{\mathrm{rx}}\left(t\right)$. For rectangular waveforms, $G_{\mathrm{rx}}$ reduces to the identity matrix, i.e., $G_{\mathrm{rx}}=I_{M}$ [38]. By using symplectic finite Fourier transform (SFFT), $Y_{\mathrm{TF}}$ is transformed to the DD domain to form $Y_{\mathrm{DD}}\in C^{M\times N}$, which is expressed as [38]

(14) $$Y_{\mathrm{DD}}=F_{M}^{H}Y_{\mathrm{TF}}F_{N}.$$

With the DD domain signal $Y_{\mathrm{DD}}$, an enhanced CE method introducing the ELM network is proposed in Section 3 to improve the CE accuracy for RIS-assisted OTFS systems. In particular, by integrating the cascaded channel $h_{\mathrm{TRR}}$ of the RIS system into the equivalent channel H in the OTFS system, the time-varying wireless channel can be transformed into a quasi-static wireless channel.

## 3. Enhanced CE by Introducing ELM Network

In this section, the proposed enhanced CE for RIS-assisted OTFS systems using the ELM network is elaborated. In Section 3.1, the pilot deployment in the DD domain is given. Then, the initial feature extraction, which is employed to enhance ELM learning, is presented in Section 3.2. With the extracted initial features, an ELM-enhanced CE (ELM-EnCE) scheme is developed in Section 3.3.

### 3.1. Pilot Placement

**Figure 2:**
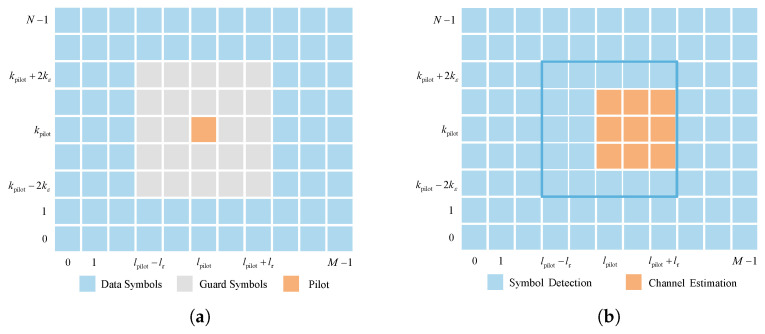
The schematic diagram of the symbols at transceiver [5]. (**a**) Symbol placement diagram at the transmitter. (**b**) The schematic diagram of received symbols.

The pilot placement scheme in [5] is employed to assist the CE in RIS-assisted OTFS systems. From [5], a single pilot symbol is embedded in the DD grids, which is guarded by a zero-valued symbol for each OTFS transmission frame in the DD domain (i.e., $X_{\mathrm{DD}}$). The necessary zero guard symbols ensure that the received symbols for channel estimation and data detection are not affected by mutual interference due to the transmission delay and Doppler shifts. The symbol placement diagram is shown in Figure 2a, where the pilot location is indexed by $\left[k_{\mathrm{pilot}},l_{\mathrm{pilot}}\right]$ with $0\leq k_{\mathrm{pilot}}\leq N-1$ and $0\leq l_{\mathrm{pilot}}\leq M-1$. Then, the transmitted symbol (i.e., the pilot symbol, guard symbols, and data symbols) on the (k,l)-th DD grid is given by [5]

(15) $$x\left[k,l\right]=\left\{\begin{array}{cc} x_{\mathrm{pilot}}, & k=k_{\mathrm{pilot}}\;\mathrm{and}\;l=l_{\mathrm{pilot}}\\ 0, & k_{\mathrm{pilot}}-2k_{\varepsilon }\leq k\leq k_{\mathrm{pilot}}+2k_{\varepsilon }\\ \; & \mathrm{and}\;l_{\mathrm{pilot}}-l_{\tau }\leq l\leq l_{\mathrm{pilot}}+l_{\tau }\\ x_{d}\left[k,l\right], & \mathrm{otherwise} \end{array}\right.,$$

where $x_{\mathrm{pilot}}$, $x_{d}\left[k,l\right]$, $l_{\tau }$, and $k_{\varepsilon }$ denote the pilot symbol, the (k,l)-th data symbol, the normalized maximum delay, and the normalized maximum Doppler shift, respectively. $l_{\tau }=\tau_{\max}M\Delta f$ and $k_{\varepsilon }=\varepsilon_{\max}NT$, where $\tau_{\max}$ and $\varepsilon_{\max}$ are the maximum delay and Doppler shift, respectively. $l_{\tau }$ and $k_{\varepsilon }$ can be determined based on prior channel statistics or through system-adaptive calibration [39,40]. In Equation (15), $\left(4k_{\varepsilon }+1\right)\left(2l_{\tau }+1\right)-1$ guard zeros are deployed. Accordingly, the overhead for pilot and guard symbols is calculated as $\frac{\left(4k_{\varepsilon }+1\right)\left(2l_{\tau }+1\right)}{MN}$. This overhead is small for typical channel models [5], since the delay and Doppler spreads are relatively limited compared to the size of the OTFS frame.

### 3.2. Initial Feature Extraction

Based on the pilot placement scheme in [5], the threshold-based method in [5] is utilized for the CE in this paper. The schematic diagram of the received signal $Y_{\mathrm{DD}}$ is shown in Figure 2b, where the entry $y_{\mathrm{DD}}\left[k,l\right]$ with $k_{\mathrm{pilot}}-k_{\varepsilon }\leq k\leq k_{\mathrm{pilot}}+k_{\varepsilon }$ and $l_{\mathrm{pilot}}\leq l\leq l_{\mathrm{pilot}}+l_{\tau }$ is used to detect the resolvable paths, and the remaining symbols on the grid are used for SD. A path detection threshold $T_{h}$ is employed to identify the resolvable paths. A smaller $T_{h}$ increases the false alarm probability, while a larger $T_{h}$ may miss detecting paths with small path gains. Hence, based on extensive experimentation, $T_{h}=3\sigma_{p}$ with $\sigma_{p}^{2}$ being the effective noise power of the pilot signal is considered. This consideration is employed to provide a favorable trade-off between false detection and miss detection probability [5]. By denoting the index of the *p*-th (p=1,2,⋯,P) resolvable path on the DD grids as $\left({\tilde{k}}_{p},\;{\tilde{l}}_{p}\right)$, its path gain is estimated as

(16) $${\hat{h}}_{\mathrm{DD}}\left[{\tilde{k}}_{p},{\tilde{l}}_{p}\right]\;=\;\frac{y_{\mathrm{DD}}\left[{\tilde{k}}_{p},{\tilde{l}}_{p}\right]}{x_{\mathrm{pilot}}},\;\mathrm{if}\;\left|y_{\mathrm{DD}}\left[{\tilde{k}}_{p},{\tilde{l}}_{p}\right]\right|\geq T_{h}.$$

With the estimated ${\hat{h}}_{\mathrm{DD}}\left[{\tilde{k}}_{p},{\tilde{l}}_{p}\right]$ in Equation (16), the equivalent channel in the DD domain, denoted as $\hat{H}\in C^{MN\times MN}$, is given by [36]

(17) $$\hat{H}=\sum_{p=1}^{P}{\hat{h}}_{\mathrm{DD}}\left[{\tilde{k}}_{p},{\tilde{l}}_{p}\right]\Pi^{{\tilde{l}}_{p}}\Delta^{{\tilde{k}}_{p}}.$$

Threshold-based initial feature extraction is typically vulnerable to noise and interference, which significantly degrades its accuracy [40]. To address this issue, we view $\hat{H}$ as the initial feature in this paper and introduce an ELM network to improve the CE accuracy. In an ELM network, the vectorized input is usually required. Thus, the initial feature of the ELM network (denoted as $\tilde{H}\in C^{M^{2}N^{2}\times 1}$) can be obtained by vectoring $\hat{H}$ column-wise, i.e.,

(18) $$\tilde{H}=\mathrm{vec}\left(\hat{H}\right).$$

With the initial feature $\tilde{H}$, we design an ELM network in Section 3.3 to enhance the CE accuracy.

**Remark 1.**  *By integrating the cascaded channel $h_{\mathrm{TRR}}$ of the RIS system into the equivalent channel H of the DD domain in the OTFS system, the time-varying wireless channel transforms into a quasi-static wireless channel. This proves beneficial for CE in high-speed mobile communication scenarios. Consequently, based on the pilot placement scheme of [5], the threshold-based initial feature extraction effectively estimates the quasi-static channel, extracting significant channel features in the DD domain to facilitate subsequent network learning.*

### 3.3. CE Enhancement with ELM Network

The developed ELM network is presented in Algorithm 1 and its main phases are described as follows.
**Algorithm 1** The algorithm of ELM-EnCE**Input:** The received signal $Y_{\mathrm{DD}}$, the positive threshold $T_{h}$, the input weights W and biases b of the ELM, and the number of training samples, i.e., *Q*.

** Output:** The output weight of the hidden layer (i.e., Y), and the enhanced CE $H_{\mathrm{en}}$.
**Offline training:**
  1: **for** q=1,⋯,Q **do**  2:      Collect the *q*-th training sample ${\tilde{H}}_{q}$ according to the equations from Equation (16) to Equation (18).  3:      Calculate the hidden layer output $o_{q}$ according to Equation (19).  4: **end for**  5: Collect $o_{1},\cdots ,o_{Q}$ to form $O=\left[o_{1},\cdots ,o_{Q}\right]$, i.e., Equation (20).  6: Calculate the output weight of the hidden layer (i.e., Υ) by using Equation (21).
**Online deployment:**  7: By using Equations (13) and (14), we transform the received r to the DD domain to obtain $Y_{\mathrm{DD}}$.  8: Estimate ${\hat{h}}_{\mathrm{DD}}\left[{\tilde{k}}_{p},{\tilde{l}}_{p}\right]$ (i.e., the gain of the *p*-th path) according to Equation (16), i.e., the threshold-based estimation method.  9: Estimate the equivalent channel $\hat{H}$ in the DD domain by using Equation (17).
10: Vectorize $\hat{H}$ column-wise to obtain the initial feature matrix $\tilde{H}$ according to Equation (18).
11: Feed $\tilde{H}$ into the trained ELM network to obtain the enhanced CE $H_{\mathrm{en}}$.


#### 3.3.1. Network Architecture

The number of the input and output neurons is designed as $M^{2}N^{2}$ to ensure consistency with the data dimension, and the hidden neurons are configured as $2M^{2}N^{2}$ to effectively capture the input features. It should be noted that this lightweight architecture of ELM is constructed to balance the complexity with performance gain, as determined through fine-tuning of neuron sizes based on extensive experimentation [22]. Further increasing the number of neurons increases computational complexity without significant improvement in estimation accuracy, whereas reducing the number of neurons results in unsatisfactory performance. From [32], the sigmoid function is employed as the activation function, which is defined as $\sigma \left(x\right)=1/\left(1+e^{-x}\right)$. The input weights $W\in C^{2M^{2}N^{2}\times M^{2}N^{2}}$ and biases $b\in C^{2M^{2}N^{2}\times 1}$ are randomly initialized for ELM training [32]. With the designed network architecture, the ELM network learns the output weight matrix by offline training.

#### 3.3.2. Offline Training

The training dataset consists of *Q* samples, denoted as $\left\{\left({\tilde{H}}_{q},{\tilde{H}}_{\mathrm{Label},q}\right),q=1,2,\cdots ,Q\right\}$ with ${\tilde{H}}_{q}$ and ${\tilde{H}}_{\mathrm{Label},q}$ being the training data and training label of the *q*-th sample, respectively. The training set of the ELM is generated through Equation (16) to Equation (18). In this paper, the number of training samples is set as $Q=10^{4}$ [32]. With the *q*-th training sample $\left({\tilde{H}}_{q},{\tilde{H}}_{\mathrm{Label},q}\right)$, the hidden layer output, denoted as $o_{q}\in C^{2M^{2}N^{2}\times 1}$, is calculated by

(19) $$o_{q}=\sigma \left(W{\tilde{H}}_{q}+b\right).$$

By collecting $o_{1},\cdots ,o_{Q}$, we have

(20) $$O=\left[o_{1},\cdots ,o_{Q}\right],$$

where $O\in C^{2M^{2}N^{2}\times Q}$ is employed to form the output weight matrix of the hidden layer. By denoting this output weight matrix as $Y\in C^{M^{2}N^{2}\times 2M^{2}N^{2}}$, we have [32]

(21) $$Y={\tilde{H}}_{\mathrm{Label}}O^{H},$$

where ${\tilde{H}}_{\mathrm{Label}}=\left[{\tilde{H}}_{\mathrm{Label},1},{\tilde{H}}_{\mathrm{Label},2},\cdots ,{\tilde{H}}_{\mathrm{Label},Q}\right]$ with ${\tilde{H}}_{\mathrm{Label}}\in C^{M^{2}N^{2}\times Q}$.

#### 3.3.3. Online Deployment

According to Equations (13) and (14), the received r given in Equation (12) is transformed into the DD domain to obtain $Y_{\mathrm{DD}}$. By using the threshold-based estimation, the path gain ${\hat{h}}_{\mathrm{DD}}\left[{\tilde{k}}_{p},{\tilde{l}}_{p}\right]$ is obtained according to Equation (16). Then, the DD domain equivalent channel matrix $\hat{H}$ is generated according to Equation (17). By using Equation (18), the generated $\hat{H}$ is vectorized column-wise to form $\tilde{H}$. Finally, we view $\tilde{H}$ as the initial network feature and use the ELM network to enhance the CE. By denoting the enhanced estimation as $H_{\mathrm{en}}\in C^{M^{2}N^{2}\times 1}$, we have

(22) $$H_{\mathrm{en}}=Y\;\cdot \;\sigma \left(W\tilde{H}+b\right).$$

With the enhanced CE (i.e., $H_{\mathrm{en}}$), the SD algorithm (e.g., MP algorithm in [6]) is employed to recover the data symbol, which benefits from the enhanced CE as well.

**Remark 2.**  *The threshold-based initial feature extraction method presented in [5] does not consider the denoising processing, degrading the CE accuracy. This inaccuracy of CE decreases subsequent SD performance. Therefore, the ELM network developed in this paper can be viewed as a denoising network. The initial feature extraction and CE enhancement with the ELM network mutually complement each other. On one hand, the single hidden layer of the ELM network structure imposes limitations on its learning ability. Leveraging the threshold-based feature extraction in [5] as the preprocessing facilitates ELM learning by focusing solely on denoising significant path features. On the other hand, the threshold-based channel estimation from [5] also benefits from the denoising capabilities of the ELM network. With offline training, the ELM captures noise and interference distributions, providing valuable statistical insights to enhance the estimation accuracy of the complex gains on significant paths.*

## 4. Results and Discussion

In this section, we validate the effectiveness and robustness of the proposed CE method by numerical simulations. In Section 4.1, the basic parameters involved in the simulations are given. Then, we verify the effectiveness of ELM-EnCE for RIS-assisted OTFS systems in Section 4.2. The robustness analysis against parameter impacts is presented in Section 4.3.

### 4.1. Parameter Settings

The basic parameters involved in the simulations are given as follows: M=16, N=8, and R=8 [32,41]. The carrier frequency $f_{c}$ and the subcarrier interval Δf are set as $f_{c}=4\;\mathrm{GHz}$ and Δf=15kHz, respectively. The modulation order, denoted as *I*, is set to I=4, and 4-quadrature amplitude modulation (4-QAM) is adopted. The average pilot and data signal-to-noise ratios (SNRs) are denoted as ${\mathrm{SNR}}_{p}=\frac{|x_{\mathrm{pilot}}{|}^{2}}{\sigma^{2}}$ and $\mathrm{SNR}=\frac{E\left(|x_{d}{|}^{2}\right)}{\sigma^{2}}$, respectively. According to [5], we use $\sigma_{p}^{2}=\frac{1}{{\mathrm{SNR}}_{p}}$ to represent the effective noise power of the pilot signal. For all the simulations, $\sigma^{2}=1$ is assumed for simplicity [5]. We employ the standard Extended Vehicular A (EVA) model as the composite channel model [42] in these simulations, considering a speed of 300 km/h (i.e., *v* = 300 km/h). The Doppler shift corresponding to the *p*-th tap is generated by Jake’s spectrum and is defined as $\varepsilon_{p}=\varepsilon_{\max}\cos(\varphi_{p})$, where $\varepsilon_{\max}$ is the maximum Doppler shift with $1.1\times 10^{3}$ Hz and $\varphi_{p}$ is uniformly distributed over $\left(0,2\pi \right]$. For data detection, we employ the MP algorithm in [6], with the number of iterations set to 15. The given ELM network is trained and tested on an Intel Xeon(R) E5-2620 CPU 2.1GHz 16 server. The training process of the proposed ELM network is conducted offline using training samples of simulated channel realizations. All simulation results are the average over 10000 Monte Carlo runs.

For expression simplicity, we use the following notations: “OTFS” for the baseline method in [6], “RIS OTFS” for the proposed method without ELM, “OTFS with ELM” for the ELM-enhanced version of [6], “ReEsNet” and “Channelformer” for the respective deep residual learning and attention-based DL baselines, and “Prop” for the proposed ELM-EnCE method. For the network training, the model of “ReEsNet” is trained with a minibatch size of 128 for 100 epochs, where the Adam optimizer is employed with learning rate 0.001 [43]. Correspondingly, the minibatch size and epochs of “Channelformer” are set as 128 and 100, respectively. The Adam optimizer is employed with an initial learning rate of 0.002 and reduced by a drop factor of 0.5 every 50 epochs [44].

### 4.2. Effectiveness Analysis

**Figure 3:**
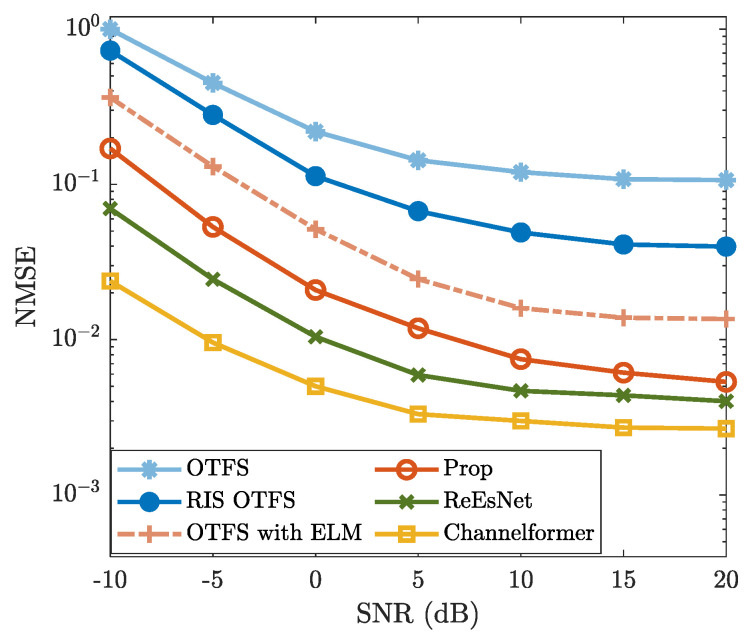
NMSE vs. SNR, where M=16, N=8, R=8, $f_{c}=4$ GHz, Δf=15kHz, I=4, and v=300km/h are considered.

**Figure 4:**
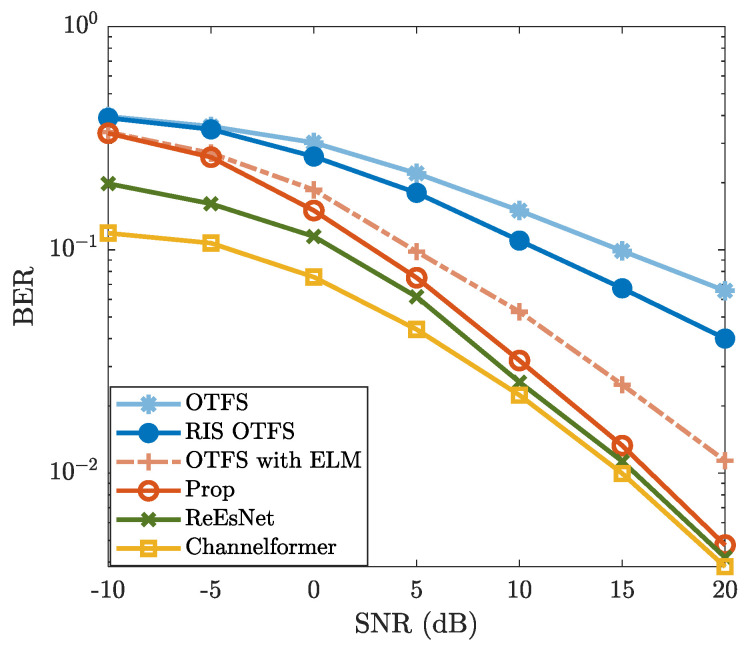
BER vs. SNR, where M=16, N=8, R=8, $f_{c}=4$ GHz, Δf=15kHz, I=4, and v=300km/h are considered.

To validate the effectiveness of the proposed method, Figure 3 and Figure 4 show the BER and NMSE performance. From Figure 3, the NMSE of “Prop” is consistently lower than those of “OTFS”, “RIS OTFS”, and “OTFS with ELM” for each specific SNR. This indicates that “Prop” is effective in improving the CE accuracy of “OTFS”, “RIS OTFS”, and “OTFS with ELM”, thereby enhancing their NMSE performance. Notably, the NMSE of “Prop” is lower than that of “RIS OTFS” for each given SNR. Similarly, the NMSE of “OTFS with ELM” is also lower than that of “OTFS” for simulated SNR regions. These results validate the effectiveness of introducing the ELM network. In other words, methods incorporating the ELM network demonstrate improved NMSE performance compared to those without ELM. Figure 3 also demonstrates that the NMSE performance of “Prop” is slightly inferior to that of the DL-based methods “ReEsNet” and “Channelformer”. However, both “ReEsNet” and “Channelformer” require intricate parameter tuning and prolonged training time, which are not necessary for the proposed method. More importantly, the NMSE curves of “ReEsNet” and “Channelformer” exhibit a performance floor in high-SNR regions (e.g., ≥10 dB), indicating limited performance gains. Therefore, with a much simpler implementation, the proposed method achieves acceptable NMSE performance and holds significant potential compared to DL-based CE methods.

In Figure 4, the BER performance of “Prop” exhibits a similar trend as observed in Figure 3. The proposed method achieves significantly lower BER compared to “OTFS”, “RIS OTFS”, and “OTFS with ELM”, while remaining slightly inferior to the DL-based “ReEsNet” and “Channelformer”, particularly in the high-SNR region (e.g., ≥10 dB). The BER performance of “OTFS”, “RIS OTFS”, and “OTFS with ELM” is improved due to enhanced CE accuracy by leveraging the ELM network. Although the proposed method does not outperform “ReEsNet” and “Channelformer”, it demonstrates comparable performance at high SNRs. Moreover, it offers notable advantages in terms of training simplicity and computational efficiency. On the whole, both Figure 3 and Figure 4 validate the effectiveness of the proposed method and highlight the great potential of ELM-based CE.

**Figure 5:**
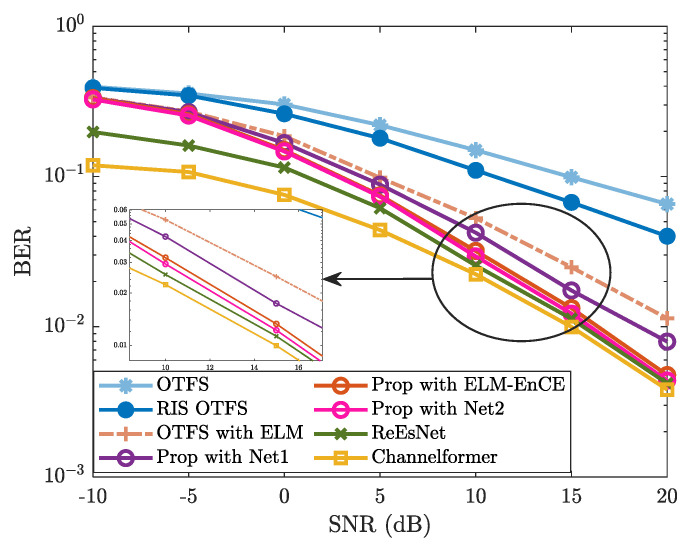
BER vs. SNR against the impact of network structure, where M=16, N=8, R=8, $f_{c}=4$ GHz, Δf=15kHz, I=4, and v=300km/h are considered.

To further verify the effectiveness and advantages of the proposed ELM network, we analyze the BER performance of the proposed method against the impact of network structure. Figure 5 shows the BER performance of “Prop” using different ELM structures, denoted by “Net1” and “Net2”. Correspondingly, their network structures are presented in Table 1. From Figure 5, the BER of “Prop with ELM-EnCE” (i.e., the proposed method) is obviously lower than that of “Prop with Net1”, particularly in the SNR region of 5dB to 20dB. Compared with “Prop with Net2”, the proposed method exhibits a slightly higher BER in regions with relatively high SNR (e.g, 10dB≤SNR≤20dB), and remains almost consistent in regions with relatively low SNR. According to Table 1 and Figure 5, by further reducing the number of hidden neurons of the ELM, a more lightweight network (i.e., Net1) is formed with obvious degradation in CE accuracy and subsequent BER performance. Instead, using a more complex ELM network (i.e., Net2) with an increased number of hidden neurons does not yield significant BER improvements, while further increasing the computational complexity. To conserve computational resources while still ensuring satisfactory CE accuracy, we adopt the current network architecture as described in Section 3.3.1.

**Figure 6:**
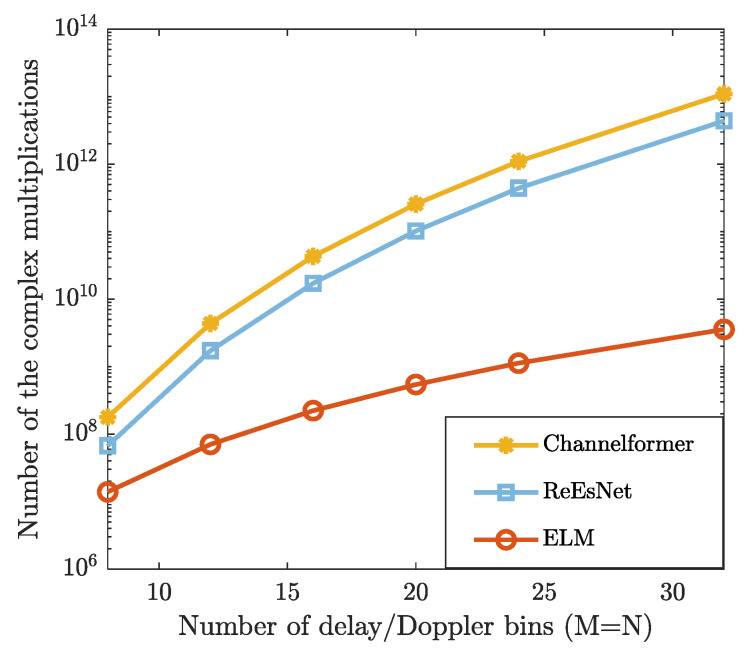
The number of the complex multiplications is evaluated under the different numbers of the OTFS frame size.

In Figure 6, we compare the complexity of the proposed method with that of DL-based methods (e.g., “ReEsNet” and “Channelformer”) in terms of the number of complex multiplications. It can be observed that the proposed ELM method consistently requires fewer complex multiplications than both “ReEsNet” and “Channelformer” for each given frame size. Considering the comparisons in NMSE and BER performance, the proposed ELM network achieves a better trade-off between the complexity and the estimation accuracy. Additionally, the average running time of each method is provided in Table 2. From Table 2, the proposed ELM method has significantly shorter running time compared to other comparison methods, demonstrating its superiority.

### 4.3. Robustness Analysis

In this subsection, we discuss the robustness of the proposed method against the impacts of modulation order *I* (of QAM), UE speed *v*, and the number of sub-surfaces *R*, respectively. With the exception of the parameters discussed in the robustness analysis, other basic parameters remain the same as those detailed in Section 4.1.

#### 4.3.1. Robustness Against *I*

**Figure 7:**
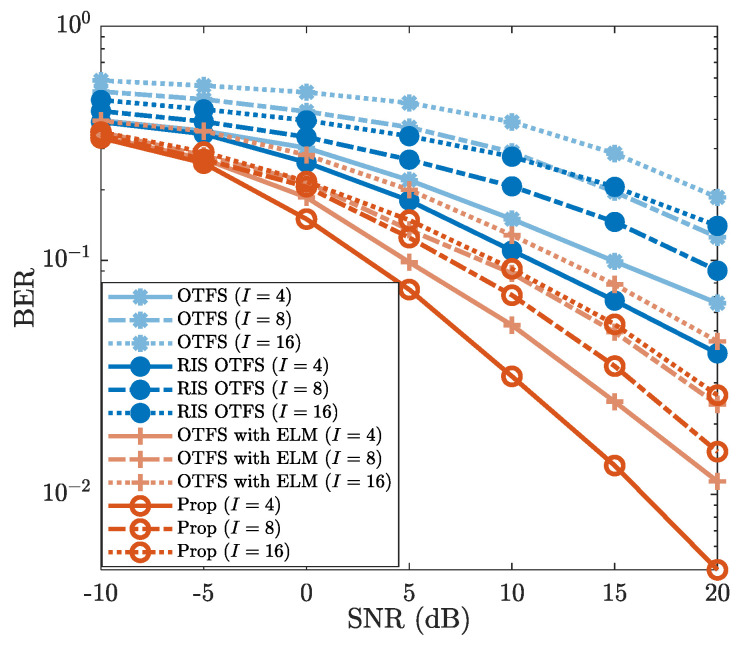
BER vs. SNR, where I=4, I=8, and I=16 are considered.

In Figure 7, we illustrate the robustness of the proposed method against the impact of the modulation order *I*, where I=4, I=8 and I=16 are considered. From Figure 7, it is evident that BERs increase as the modulation order *I* increases. For example, when SNR is 10dB, the “Prop” employing 4-QAM (i.e., I=4) exhibits a BER of $3.2\times 10^{-2}$, while the BERs of 8-QAM (I=8) and 16-QAM (I=16) are about $7.1\times 10^{-2}$ and close to $10^{-1}$, respectively. Furthermore, Figure 7 illustrates that the “Prop” achieves a lower BER than those of “OTFS”, “RIS OTFS”, and “OTFS with ELM” for each modulation order *I*. For the case where I=4 (4-QAM) and SNR is 20dB, the BER of “Prop” is about $4\times 10^{-3}$, whereas the BERs of “OTFS”, “RIS OTFS”, and “OTFS with ELM” all exceed $10^{-2}$. Despite the variation of modulation order *I*, the BER performance of the proposed method consistently outperforms those of “OTFS”, “RIS OTFS”, and “OTFS with ELM”. Therefore, the proposed method possesses a good robustness against the impact of the modulation order *I*.

#### 4.3.2. Robustness Against *v*

**Figure 8:**
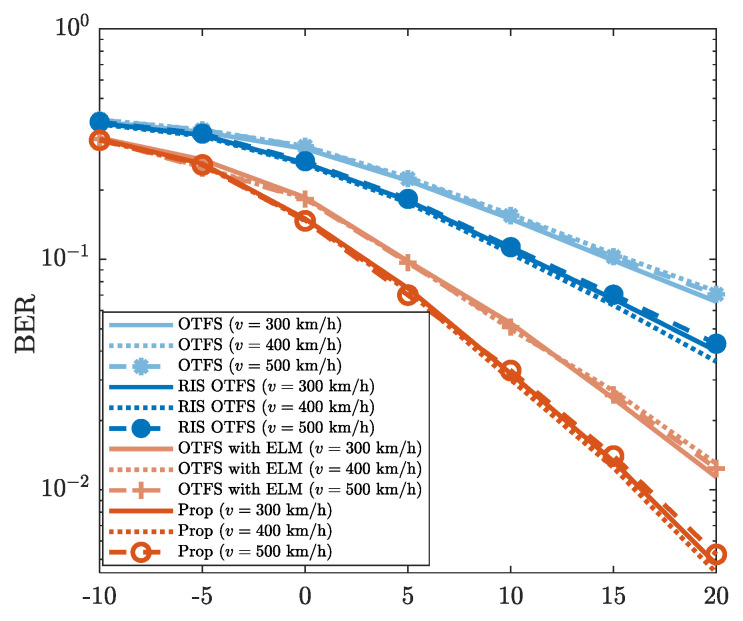
BER vs. SNR, where *v* = 300 km/h, *v* = 400 km/h, and *v* = 500 km/h are considered.

Figure 8 presents the robustness of the proposed method against varying UE speeds (i.e., *v* = 300 km/h, *v* = 400 km/h and *v* = 500 km/h). The “Prop” exhibits highly similar BER performance under different UE speeds. For example, when SNR is 10dB, the BERs of “Prop” for different *v* are all approximately $3.3\times 10^{-2}$. This confirms the BER performance reliability of the proposed method in high-mobility scenarios. Additionally, from Figure 8, it is evident that “Prop” achieves a lower BER than those of “OTFS”, “RIS OTFS”, and “OTFS with ELM”. For the case where SNR is 15dB, the BER of “Prop” is about $1.4\times 10^{-2}$, while the BERs of “OTFS”, “RIS OTFS”, and “OTFS with ELM” are all higher than $10^{-2}$. This illustrates that the BER performance of “Prop” outperforms those of “OTFS”, “RIS OTFS”, and “OTFS with ELM” for different UE speeds. On the whole, the proposed method improves the BER performance of “OTFS”, “RIS OTFS”, and “OTFS with ELM”, even against the variations of the UE speed *v*.

#### 4.3.3. Robustness Against *R*

**Figure 9:**
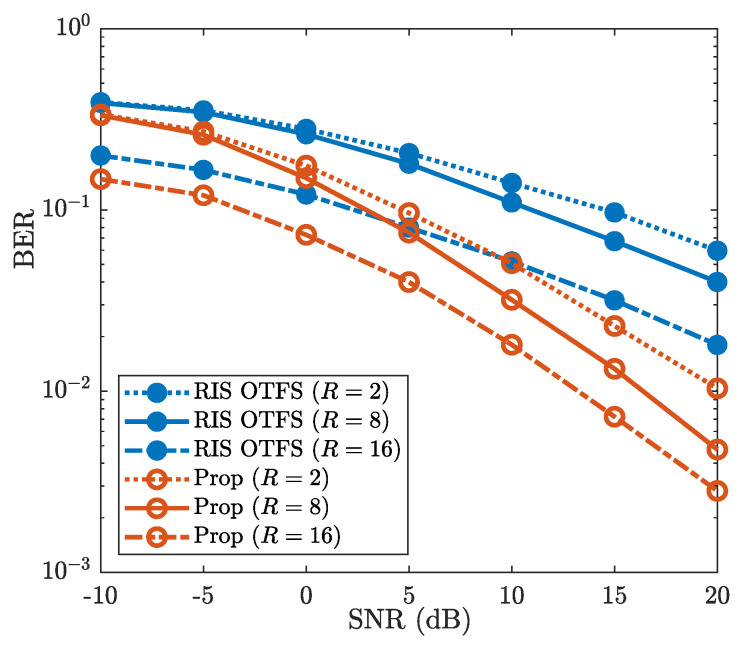
BER vs. SNR, where R=2, R=8, and R=16 are considered.

Figure 9 demonstrates the robustness of the proposed method for different numbers of sub-surfaces, where R=2, R=8 and R=16 are considered. It can be observed that the BERs decrease as the number of sub-surfaces *R* increases. For example, when SNR is 20dB, the BERs of “Prop” are $1.1\times 10^{-2}$, $4.7\times 10^{-3}$ and $2.8\times 10^{-3}$ for R=2, R=8 and R=16, respectively. This validates the benefits of increased RIS sub-surfaces on BER performance improvement. Additionally, Figure 9 shows that the “Prop” achieves lower BER than that of “RIS OTFS” for each given value of *R*. When R=16 and SNR is 10dB, the BER of “Prop” is about $1.8\times 10^{-2}$, while the BER of “RIS OTFS” reaches about $5\times 10^{-2}$. For the number of different sub-surfaces *R*, the “Prop” consistently provides better BER performance than that of “RIS OTFS”. Therefore, against the varying number of sub-surfaces *R*, the proposed method achieves superior BER performance compared to “RIS OTFS”.

## 5. Conclusions

In this paper, we have investigated an ELM-EnCE method to improve the CE accuracy in RIS-assisted OTFS systems. Recognizing the inherent limitations of ELM, such as insufficient network parameters compared to DL networks, the paper introduces a threshold-based method to extract initial features. The initial features are utilized to enhance ELM learning ability, leading to an improved CE accuracy. This improved CE accuracy consequently benefits subsequent SD performance. Experimental results demonstrate that the proposed method effectively enhances SD performance and exhibits robustness against modulation order and UE speed impacts. Additionally, exploring the robustness of the proposed method under non-simulated (such as test-time distributional shifts, hardware imperfections and distortions, and noise-corrupted) training samples and extending it to multiple antennas and multi-user communication systems can further enhance its practical applicability.

## Figures and Tables

**Table 1:** The structure of the comparison networks.

Net1			Net2		
Layer	Neuron Size	Activation Function	Layer	Neuron Size	Activation Function
Input	$M^{2}N^{2}$	Linear	Input	$M^{2}N^{2}$	Linear
Hidden	$M^{2}N^{2}$	Sigmoid	Hidden	$6M^{2}N^{2}$	Sigmoid
Output	$M^{2}N^{2}$	Linear	Output	$M^{2}N^{2}$	Linear

**Table 2:** Average running time of the compared methods (in seconds).

Channelformer	ReEsNet	ELM
1835	1176	58

## Data Availability

The original contributions presented in this study are included in the article. Further inquiries can be directed to the corresponding author.
